# Arachidonic acid pathway alterations in cerebrospinal fluid of dogs with naturally occurring spinal cord injury

**DOI:** 10.1186/s12868-016-0269-4

**Published:** 2016-06-10

**Authors:** Rae L. Russell, Jonathan M. Levine, Nick D. Jeffery, Colin Young, Armando Mondragon, Bryan Lee, C. Elizabeth Boudreau, C. Jane Welsh, Gwendolyn J. Levine

**Affiliations:** Department of Small Animal Clinical Sciences, College of Veterinary Medicine and Biomedical Sciences, Texas A&M University, College Station, TX 77843 USA; Department of Veterinary Integrative Biosciences, College of Veterinary Medicine and Biomedical Sciences, Texas A&M University, College Station, TX 77843 USA; Department of Veterinary Clinical Sciences, College of Veterinary Medicine, Iowa State University, 1720 Veterinary Medicine, Ames, IA 50011 USA; Department of Veterinary Pathobiology, College of Veterinary Medicine and Biomedical Sciences, Texas A&M University, TAMU 4467, College Station, TX 77843 USA

**Keywords:** Canine, Neurotrauma, Prostaglandin E2, Phospholipase A2

## Abstract

**Background:**

Canine intervertebral disc πherniation causes a naturally-occurring spinal cord injury (SCI) that bears critical similarities to human SCI with respect to both injury pathomechanisms and treatment. As such, it has tremendous potential to enhance our understanding of injury biology and the preclinical evaluation of novel therapies. Currently, there is limited understanding of the role of arachidonic acid metabolites in canine SCI.

**Results:**

The CSF concentrations of PLA2 and PGE2 were higher in SCI dogs compared to control dogs (p = 0.0370 and 0.0273, respectively), but CSF LCT4 concentration in SCI dogs was significantly lower than that in control dogs (p < 0.0001). Prostaglandin E2 concentration in the CSF was significantly and positively associated with increased severity of SCI at the time of sampling (p = 0.041) and recovery 42 days post-injury (p = 0.006), as measured by ordinal behavioral scores.

**Conclusion:**

Arachidonic acid metabolism is altered in dogs with SCI, and these data suggest that these AA metabolites reflect injury severity and recovery, paralleling data from other model systems.

## Background

Several experimental animal models of spinal cord injury (SCI) have been established, including contusion, laceration, clip compression, and crush in a variety of species [1–3]. These systems generate highly stereotypical injuries and minimize heterogeneity in severity, timing of injury, genetic background, and environmental exposure. While elimination of inter-animal variability likely enhances detection of the effects of putative therapeutic interventions, it does not fully reflect the diverse injury characteristics that complicate naturally-occurring SCI [4].

Canine intervertebral disc herniation (IVDH) causes a naturally-occurring form of SCI that bears critical similarities to human SCI with respect to both injury pathomechanisms and treatment. The resulting SCI occurs spontaneously, consists of varying components of compression and contusion, and is treated with a combination of decompressive surgery and physical rehabilitation [5, 6]. Histologic facets of injury parallel those detected in both humans with SCI and SCI models, including axon destruction demyelination, and centrally-oriented necrosis/cavitation [7]. Spinal cord lesions in affected dogs contain activated microglia [8]; have aberrantly increased expression of IL-6, IL-8 [9], and matrix metalloproteinase-9 (MMP-9) [10]; and contain a population of peripherally-derived leukocytes [8, 11]. Additionally, these inflammatory events result in loss of blood-spinal cord barrier integrity and increased oxidative stress [12]. The similarities between human SCI and canine SCI resulting from IVDH have prompted the development of validated outcome measures including ordinal gait scores, kinematics, kinetics, urodynamics, and sensory testing in order to detect subtle improvement associated with experimental therapeutic interventions [13–15]. Furthermore, studies by several independent groups have utilized dogs with IVDH as a second species to evaluate neuroprotective and potential regenerative strategies headed for human clinical trials [5, 14, 16, 17].

The arachidonic acid (AA) pathway is a critical mediator of secondary SCI and an attractive target for pharmacologic interventions. Metabolism of AA is ubiquitous; this polyunsaturated fatty acid is released from cell membranes by phospholipase A2 (PLA2, for review see Schaloske and Dennis [18]). In rodent spinal cord contusion models, PLA2 protein expression is induced within minutes of injury, persists for up to 7 days post-injury, and correlates with the development of demyelination and neuronal necrosis [19]. Following its release from the cell membrane, AA is metabolized into leukotrienes (LTs) and prostaglandins (PGs) via 5-lipoxygenase (5-LOX) and cyclooxygenase (COX), respectively [20, 21]. Leukotrienes and PGs function as immune cell chemoattractants, vasodilators, inducers of oxidative stress, and modulators of neurosensory processing. Further, LTs and PGs are increased acutely after experimental SCI and remain aberrantly elevated for months post-trauma [22]. Chronic dysregulation of 5-LOX and COX pathways following experimental spinal cord contusion results in depletion of lipid metabolites, altered amino acid biosynthesis, and pro-inflammatory events. Limited investigation of these pathways has occurred in large animal models of SCI. In one study that utilized an experimental canine model of compression/contusion, LTs and PGs were increased within the cerebrospinal fluid (CSF) 1 day following injury and remained elevated for approximately 7 days after the primary event [23].

In this study, we assessed alterations of AA metabolism after SCI in dogs with IVDH by using enzyme-linked immunosorbent assays (ELISA) to measure CSF concentrations of PLA2, leukotriene C4 (LTC4) and prostaglandin E2 (PGE2). We selected these mediators because they represent critical nodes in the AA pathway that are altered in experimental SCI. Our primary objective in this exploratory study was to determine if there were higher concentrations of AA metabolites in the CSF of dogs with SCI than in healthy control dog CSF. Our secondary objective was to determine if, in dogs with SCI, CSF AA pathway metabolites were correlated with functional deficits at the time of sampling and 42-day post-injury recovery as measured by a validated ordinal score.

## Methods

### Sample size determination

Sample size was determined a priori and was based on previous studies that examined inflammatory mediators (e.g., IL-8, C-reactive protein, MMP-9) in the CSF of dogs with IVDH-associated SCIs compared to CSF of healthy control dogs [24, 25]. In those studies, samples from 8 to 21 healthy controls and 35–47 dogs with IVDH were used to identify significant inter-group differences. Based on these data, we elected to utilize all control (n = 21) and SCI samples (n = 44) available within our biobank.

### Inclusion and exclusion criteria

A repository of CSF aliquots collected from the cerebellomedullary cistern, stored at −80 °C, and housed at Texas A&M University since December 2009 was screened in February 2014 for samples from dogs with IVDH-associated SCI that met the following inclusion criteria: (1) lesion between T3 and L5 vertebrae; (2) neurologic impairment of <7 days duration; (3) surgical decompression of IVDH with post-operative rehabilitation; and (4) complete medical records including neurologic score at admission and follow-up scoring at day 42 post-surgery. Dogs that were part of on-going clinical trials or had a myelogram performed as part of pre-operative diagnostics were excluded from this study.

Healthy control CSF samples were collected from purpose-bred dogs with normal physical and neurologic exams, normal complete blood counts, and normal serum biochemical analysis. All CSF samples from healthy control dogs were collected and stored in the same manner as described for dogs with IVDH-associated SCI and were required to have a normal total nucleated cell count (TNCC) (<5 cells/µL) and total protein concentration (<35 mg/dL).

### Sample collection and therapeutic procedures

Procedures in dogs with naturally-occurring SCI were performed with owner consent and consisted of standard medical and surgical care. Purpose-bred dogs were obtained and used with approval from the Texas A&M University Animal Care and Use Committee (AUP 2007-115; AUP 2011-145). All studies adhered to the National Institutes of Health Guide for Care and Use of Laboratory Animals.

Dogs with SCI underwent complete physical examination, neurologic examination, complete blood count, and serum biochemistry prior to anesthesia. Data including age, gender, duration of SCI, and recent delivery of non-steroidal anti-inflammatory drugs (NSAIDs) or glucocorticoids (GCs) were collected (Table 1). Dogs were considered treated with these drugs if NSAIDs or GCs were administered within the 7-day period prior to CSF collection. Dogs were then pre-medicated with glycopyrrolate (Robinul-V, West-Ward, Eatontown, NJ, USA) and oxymorphone (Numorphan, Endo Pharmaceuticals, Chadds Ford, PA, USA) or hydromorphone (West-Ward, Eatontown, NJ, USA). Following pre-medication, dogs were induced with propofol (Rapinovet, Abbott Labs, Chicago, IL, USA) and intubated, and anesthesia was maintained with sevoflurane (SevoFlo, Abbott Labs, Chicago, IL, USA). Thoracolumbar vertebral column imaging was performed either via magnetic resonance imaging (MR) or computed tomography (CT). Cerebrospinal fluid was then collected via needle puncture of the cerebellomedullary cistern with an aliquot saved and stored at −80 °C for further analysis. Following CSF collection and diagnostic imaging, a hemilaminectomy was performed to remove herniated, compressive intervertebral disc material and associated hemorrhage from the epidural space. Either gross appearance of the disc material or histopathology was used to confirm the diagnosis of IVDH.

**Table 1:** Population characteristics for SCI and control dogs

Variable	SCI dogs (n = 44)	Healthy control dogs (n = 21)
*Dogs*		
Median age	5.75 years	3 years
MFS at admission	2.5	N/A
TSCIS at admission	6	N/A
Injury to anesthesia time	36.5 h	N/A
*Sex characteristics*		
Female intact	4 (9 %)	0 (0 %)
Female spayed	18 (41 %)	3 (14 %)
Male intact	8 (18 %)	5 (24 %)
Male neutered	14 (32 %)	13 (62 %)
*Breeds*		
Dachshund	34 (76 %)	0 (0 %)
Labrador retriever	0 (0 %)	7 (33 %)
Mixed breed	4 (9 %)	5 (24 %)
*Injuries*		
T12–13	6 (14 %)	N/A
T13–L1	13 (30 %)	N/A
L1–L2	5 (11 %)	N/A
L2–L3	8 (18 %)	N/A
Other thoracic	8 (18 %)	N/A
Other lumbar	4 (9 %)	N/A
*Treatments*		
Non-steroidal anti-inflammatory drugs	17 (39 %)	0 (0 %)
Glucocorticoids	14 (32 %)	0 (0 %)
Both	3 (<1 %)	0 (0 %)

Following surgery, dogs were recovered and provided intravenous fentanyl citrate (Hospira Inc., Lake Forest, IL, USA) analgesia and bladder evacuation if unable to voluntarily void. Twenty-four hours later, physical rehabilitation consisting of supported overland walking, passive range of motion, and standing strength exercises were initiated. Dogs were released to their owner’s care after pain control was achieved via oral analgesics (tramadol hydrochloride, Amneal Pharaceuticals, Hauppauge, NY, USA) and urine could be voluntarily voided or the bladder manually expressed. The owners continued physical rehabilitation exercises for 6 weeks post-operatively.

### Neurologic scoring

Two separate ordinal SCI scores were used in this study, and were applied at initial evaluation and at a 42-day post-SCI re-evaluation. The modified Frankel score (MFS), and the Texas Spinal Cord Injury Score (TSCIS) have both been validated previously in dogs with IVDH-associated SCI and have been shown to have excellent inter-rater agreement, correlate well with MRI-based measures of SCI, and predict 42-day post-SCI motor outcome [26]. For both assessment tools, dogs were considered ambulatory if they could rise unassisted and take ten or more steps without falling. Dogs that were non-ambulatory had pelvic limb movements evaluated using tail support. Postural reaction scores were determined by supporting the dog in a standing position and placing the dorsum of the paw in contact with the ground. Conscious perception of mild and severe stimuli was evaluated by pinching the interdigital webbing and clamping the nail bed with hemostats, respectively. Pain sensation was considered intact based on demonstration of a behavioral (e.g., orienting to the stimulus, vocalization) or physiological (e.g., tachycardia, tachypnea) response to stimulation.

The MFS was used as a coarse ordinal system to stratify injured dogs into groups that parallel those in the American Spinal Cord Injury Association Impairment Scale (ASIA). The MFS consists of six strata where 0 = paraplegic with absent pain sensation from the hindquarters; 1 = paraplegic with pain sensation intact to severe stimuli; 2 = parapaplegic with intact sensation for mild stimuli; 3 = non-ambulatory paraparetic; 4 = ambulatory paraparetic; and 5 = signs consistent with spinal pain only.

The TSCIS was developed as a more refined system than the MFS and was used for all analyses that did not require stratification into broad functional categories. With this system, individual limbs are assessed independently and given a score based on sensation, gait, and proprioceptive placing. Sensation was scored as 0 = absent, 1 = sensation present to severe stimuli, but absent for mild stimuli, and 2 = sensation intact. Proprioceptive placing was scored as 0 when absent, 1 when delayed (correction to normal posture taking >2 s), and 2 when considered normal. For gait assessment, scores ranged from 0 to 6 for each limb as follows: 0 = no voluntary movement present when supported; 1 = intact limb protraction with no ground clearance; 2 = intact limb protraction with inconsistent ground clearance; 3 = intact limb protraction with consistent ground clearance; 4 = ambulatory with moderate paresis/ataxia (will fall occasionally); 5 = ambulatory with mild paresis/ataxia (does not fall even on slick surfaces); and 6 = normal gait.

### Measurement of AA pathway metabolite concentrations

Cerebrospinal fluid concentrations of PLA2, LTC4, and PGE2 were measured using commercially available ELISA (MyBioSource, San Diego, CA). The PLA2 ELISA (for the lipoprotein-associated isoform), catalog # MBS015390, was performed following the manufacturer’s protocol, using a standard curve ranging from 800 to 25 ng/mL. The LTC4 and PGE2 ELISAs (catalog #MBS013956 and MBS705363, respectively) were also performed following the manufacturer’s protocol, and the standard curves used were 31.2–1000 pg/mL for LTC4 and 31.25–2000 pg/mL for PGE2. All control CSF samples were run as technical duplicates. A single run was performed on injured dogs samples, because volume available was limited.

### Statistics

The primary objective of this study was to determine whether the concentrations of the AA pathway metabolites in CSF are associated with functional recovery status at 42 days. These relationships were explored first by using univariable linear regression with TSCIS at 42 days as the outcome (dependent variable). Multivariable linear regression was then used to examine the effects of the various possible interactions including initial injury severity, time delay between injury and sampling, NSAID and GC administration, through their inclusion as covariates. All analyses were conducted using commercially available software (Stata 11, StataCorp, College Station, TX).

Secondary objectives of this study were to explore relationships between injury and AA metabolite concentrations. We compared the CSF metabolite concentrations between control and SCI dogs using Mann–Whitney tests. Association between AA metabolite concentration and cell count in the CSF and with SCI severity at presentation were analyzed using linear regression. Figures were generated using GraphPad Prism, version 6.0 (GraphPad Software, San Diego, CA).

## Results

### Population characteristics

There were 44 dogs in the SCI group (Table 1). The median age was 5.75 years (range 1–12 years). The 3 most common breeds were dachshunds (n = 34; 76 %), mixed breeds (n = 4; 9 %), and shih tzus (n = 3; 7 %). There were 4 intact females (9 %), 18 spayed females (41 %), 8 intact males (18 %), and 14 neutered males (32 %). The median duration between the time of initial injury and CSF collection was 36 h (range 3–182 h). The median MFS before CSF acquisition was 2.5 (a score indicating non-ambulatory paraparesis; scores ranged from 0 to 5). The median TSCIS sub-scores at presentation were as follows: nociception 4 (range 0–4), proprioceptive placing 0 (range 0–3), and motor 2 (range 0–10). The most common vertebral levels at which compressive/contusive lesions were located based on MR and CT imaging included: T12–T13 (N = 6; 14 %), T13–L1 (N = 13; 30 %), L1–L2 (N = 5; 11 %), and L2–L3 (N = 8; 18 %); there were eight dogs with thoracic injuries at other levels (18 %) and four with lumbar injuries at other levels (9 %). There were 17 dogs (39 %) that received NSAIDs, 14 that received glucocorticoids (32 %), and 3 dogs (<1 %) that received both NSAIDs and glucocorticoids.

The 21 control dogs had a median age of 3 years (range 1–4). The 2 most common breeds were Labrador retriever and beagle (33 and 24 %, respectively, Table 1). There were 0 intact females, 3 spayed females (14 %), 5 intact males (24 %), and 13 neutered males (62 %). No control dogs received glucocorticoids or NSAIDs.

### CSF analysis in dogs with SCI and control dogs

In the SCI group, the median TNCC was 2 cells/µL (range 0–107 cells/µL), the median red blood cell count (RBC) was 10 cells/µL (range 0–11,005 cells/µL) and the median total protein concentration was 18 mg/dL (range 9–94 mg/dL). Twelve dogs had pleocytosis (TNCC > 5 cells/μL); of these the median percentage of neutrophils was 49 % (range 0–85 %), monocytes 24.5 % (range 0–100 %), lymphocytes 12 % (range 0–63 %), and eosinophils was 0 % (range 0–3 %). No pleocytosis was detected in control CSF samples. In the control group, the median TNCC was 0 cells/µL (range 0–2 cells/µL), the median RBC was 3 cells/µL (range 0–730 cells/µL) and the median total protein was 26 mg/dL (range 10–35 mg/dL).

### AA pathway mediators are dysregulated in the CSF of SCI dogs

**Fig. 1:**
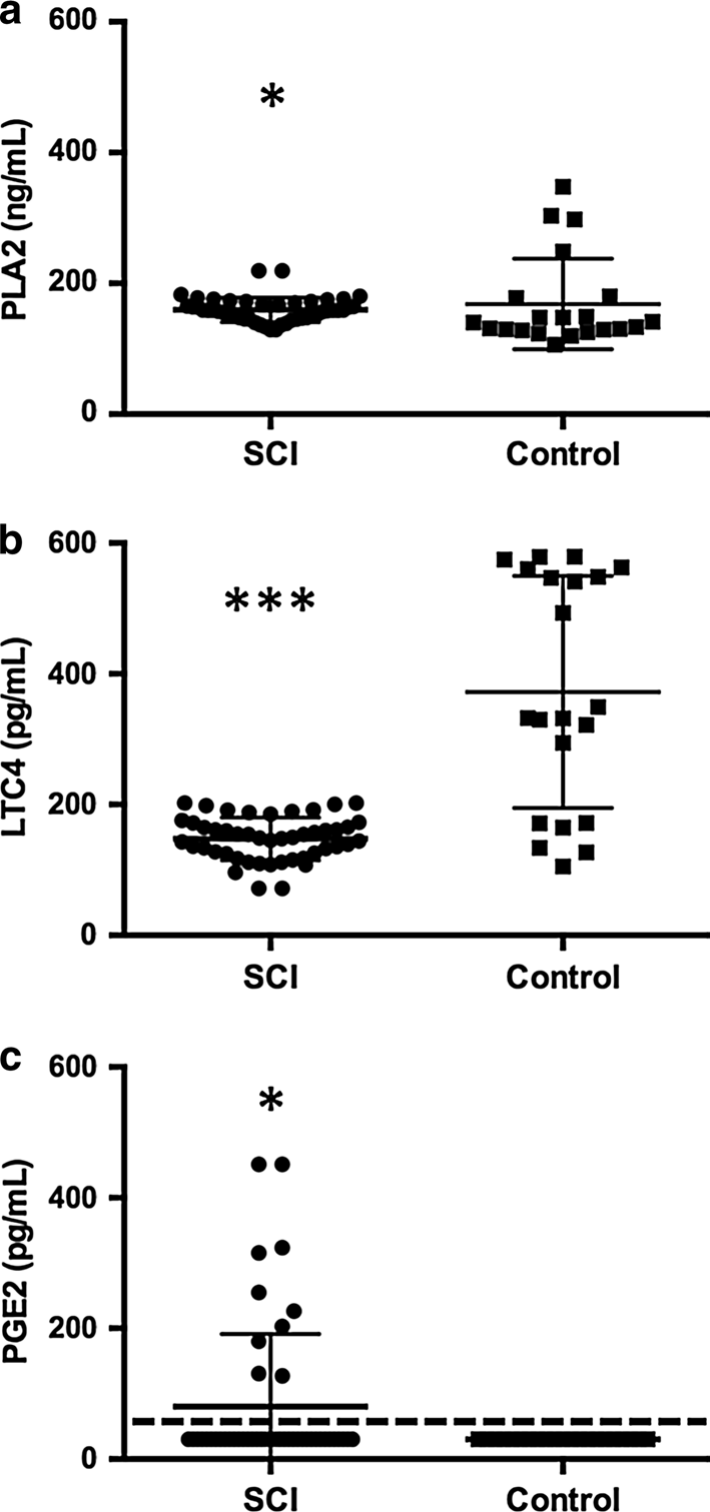
*Scatter plots* and *line* and *whisker plots* summarizing concentrations of AA metabolites in CSF of 44 dogs with SCI and 21 control dogs. There was a significantly higher CSF PLA2 concentration in dogs with SCI compared to control dogs (*asterisks*, p = 0.0370) (**a**). The concentration of LCT4 in the CSF of SCI dogs was significantly lower than that in control dogs (*asterisks*, p < 0.0001) (**b**). The concentration of PGE2 in the CSF of SCI dogs was significantly higher (**c**, *asterisks*, p = 0.0273) compared to that in control dogs (<31 pg/mL, *dotted line*)

There was significantly higher CSF PLA2 concentration (p = 0.0370) in dogs with SCI (median = 158.65 ng/mL, range 129.47–219.45 ng/mL) compared to control dogs (Fig. 1a, median = 140.08 ng/mL, range 106.04–347.93 ng/mL). The concentration of LCT4 in the CSF of SCI dogs (median = 148.69 pg/mL, range 71.88–202.37 pg/mL) was significantly lower (p < 0.0001) than that in control dogs (median = 332.27 pg/mL, range 105.49–579.09 pg/mL) (Fig. 1b). The concentration of PGE2 in the CSF of SCI dogs (median < 31 pg/mL, range <31–451.07 pg/mL) was significantly (p = 0.0273) greater compared to control dogs (Fig. 1c; all control dogs had CSF containing concentrations that were below the limit of detection for this kit <31 pg/mL).

**Fig. 2:**
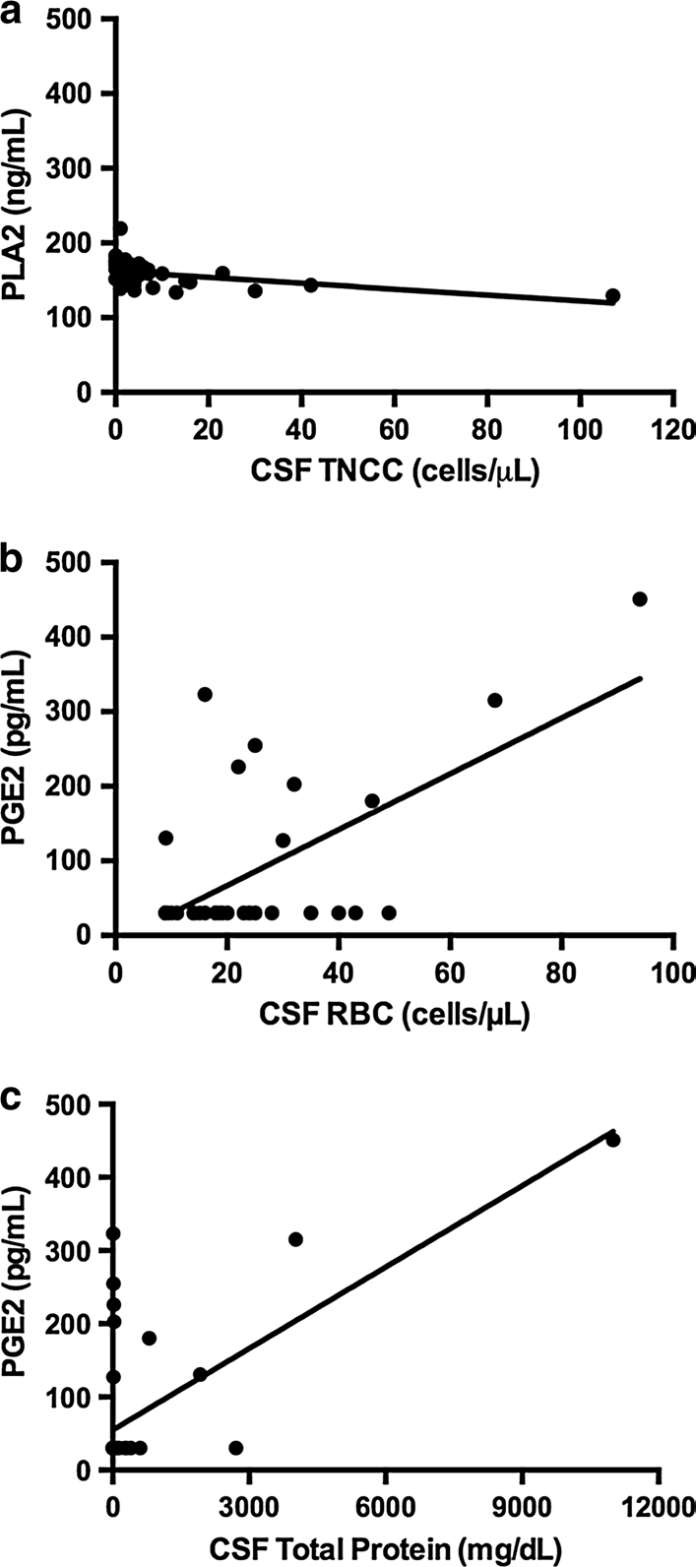
Linear regressions amongst CSF AA pathway metabolite concentrations and total nucleated cell counts (TNCC, cells/µL), CSF total protein (mg/dL), and CSF red blood cell count (cells/µL) in 44 SCI dogs. PLA2 concentration was negatively associated with TNCC in dogs with SCI (r^2^ = 0.178, slope = −0.400; p = 0.004) (**a**). CSF PGE2 concentration correlated positively with CSF total protein concentration (**b**, r^2^ = 0.422, slope = 3.75; p < 0.0001), and CSF RBC (**c**, R^2^ = 0.451, slope = 0.370; p < 0.0001)

We explored associations between CSF analytes including TNCC, RBC, total protein concentration, and percentage of leukocytes and AA pathway metabolite concentrations in the CSF of SCI dogs. The only significant associations were between PLA2 and TNCC (Fig. 2a, r^2^ = 0.178, slope = −0.400; p = 0.004), PGE2 and CSF total protein (Fig. 2b, r^2^ = 0.422, slope = 3.75; p < 0.0001), and PGE2 and CSF RBCs (Fig. 2c, r^2^ = 0.451, slope = 0.370; p < 0.0001).

### CSF PGE2 is correlated with SCI severity and 42-day outcome

**Fig. 3:**
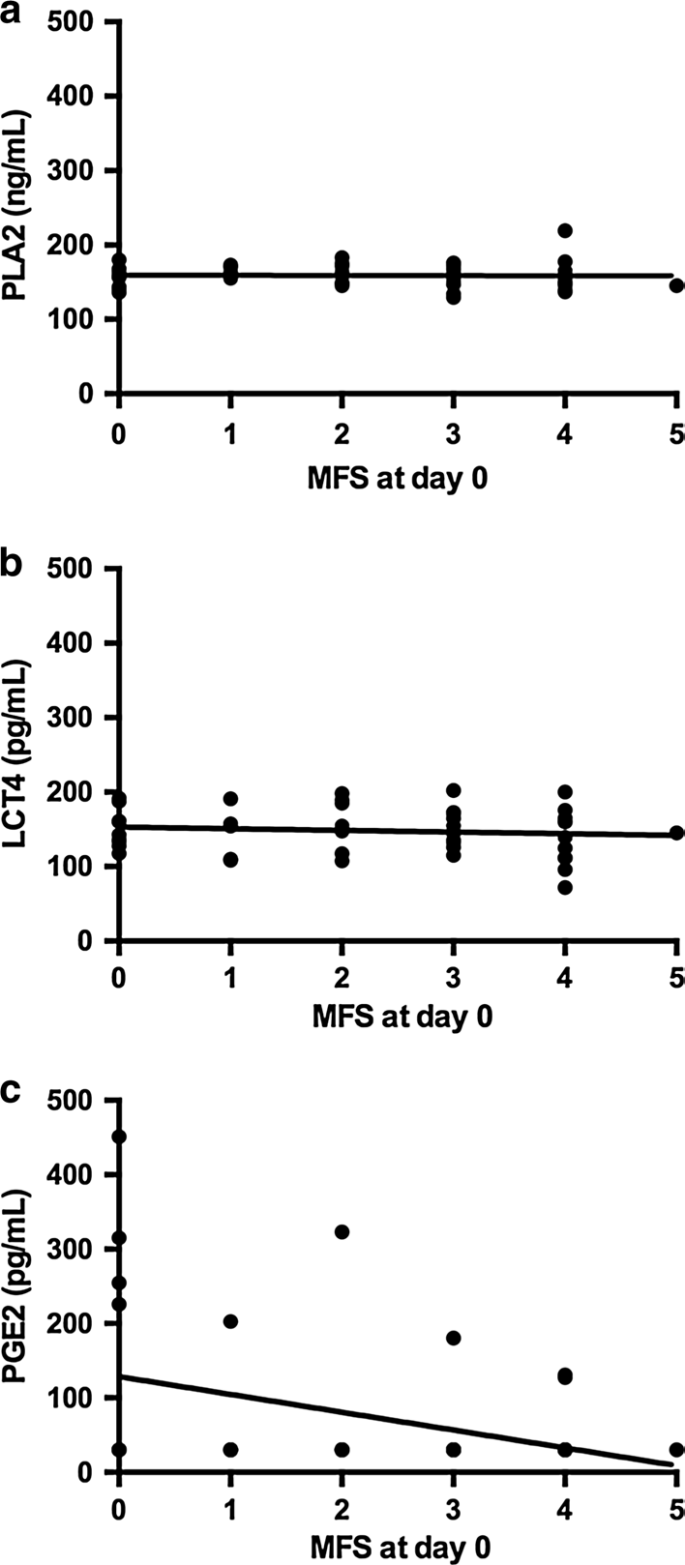
Linear regressions of CSF AA pathway metabolites and modified Frankel scores (MFS) at day of hospital admission (day 0). PLA2 and LTC4 were not significantly correlated to MFS at day 0 (r^2^ = 0.0004, slope = −0.221; p = 0.894, and r^2^ = 0.012, slope = 0.–2.23; p = 0.4756, respectively) (**a**, **b**). PGE2 was higher in SCI dogs with lower MFS at day 0 (r^2^ = 0.137, slope = − 23.9; p = 0.013) (**c**)

**Fig. 4:**
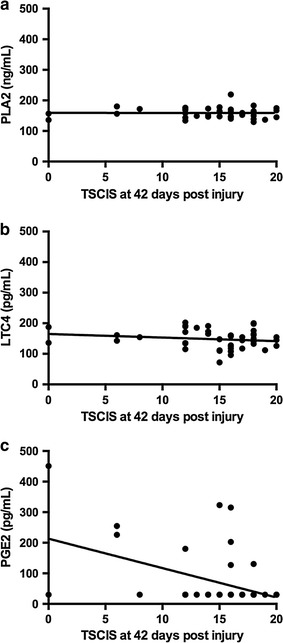
Linear regressions of AA pathway metabolites and Texas Spinal Cord Injury Scores (TSCIS) at day 42 post-injury. PLA2 and LTC4 were not significantly correlated to TSCIS at day 42 (r^2^ = 0.00003, slope = −0.021; p = 0.970, and r^2^ = 0.030, slope = 0.–1.166; p = 0.262, respectively) (**a**, **b**). PGE2 was significantly correlated to lower TSCIS at day 42 in dogs with SCI (r^2^ = 0.199, slope = −9.62; p = 0.002) (**c**)

Prostaglandin E2 concentration in the CSF was significantly and positively associated with increasing severity of SCI at the time of sampling, as measured by the MFS in univariate and multivariate models (Fig. 3c, p = 0.029 and p = 0.041, respectively). No other AA mediators were associated with SCI severity at the time of sample acquisition (Fig. 3a, b). The CSF concentrations of PLA2 and LTC4 were not significantly associated with 42 day post-SCI TSCIS (Fig. 4a, b, p = 0.970 and 0.262, respectively). Prostaglandin E2 concentration was significantly and negatively associated with 42-day post-SCI recovery as measured by the TSCIS in univariate and multivariate models (Fig. 4c, r^2^ = 0.199, slope = −9.61, p = 0.003 and p = 0.006, respectively). Because of the low number of SCI dogs in which PGE2 reached detectable concentrations we examined the sensitivity of this result to the numerous null values by repeating the test but including only the dogs with detectable values; both univariable (r^2^ = 0.39, slope = −10.47, p = 0.073) and multivariable (r^2^ = 0.51, slope = −1.77, p = 0.137) analysis revealed a non-significant association, although this may also result from the much reduced power of these tests.

## Discussion

This study broadly compared measures of AA metabolism in dogs after naturally-occurring SCI with that in healthy control dogs. The concentration of PLA2, which frees AA from phospholipid membranes, was significantly higher in SCI versus control dogs, and had a weak negative association with the total nucleated cell count in the CSF. The CSF concentration of LTC4, a pro-inflammatory leukotriene, was significantly lower in dogs with naturally-occurring SCI compared to control dogs. The CSF concentration of PGE2 was significantly higher in SCI dogs compared to control dogs, and significant relationships existed between CSF PGE2 concentration, initial SCI severity, as well as 42-day post-SCI recovery.

Here, we found CSF concentration of lipoprotein-associated PLA2 was significantly higher in SCI dogs compared to control dogs. In studies performed on rodent spinal cord homogenates post-SCI, the secretory isoform of PLA2 increases within hours of injury, is over-expressed for days following SCI, and is negatively correlated with recovery of locomotion [20]. Critically, there are 27 isoforms of PLA2, of which only 7 have been clearly demonstrated to be dysregulated in SCI [20]. We chose to measure lipoprotein-associated PLA2 because it has not been previously evaluated in the context of SCI, up-regulation has been established in neuro-inflammatory diseases such as Alzheimer’s [27], it is secreted from inflammatory cells known to be present within injured cords, and validated methodologies existed to measure it in dogs. While findings here suggest lipoprotein-associated PLA2 is released following injury, there was no association between CSF PLA2 concentration and injury severity at the time of sampling or 42-day post-SCI outcome. Additionally, CSF PLA2 concentration was weakly, but negatively, associated with CSF TNCC. The complex and overlapping role of PLA2 isoforms, treatment of dogs with immune-modulating drugs, and sample size may explain our inability to detect associations between CSF lipoprotein-associated PLA2 concentrations and certain facets of injury.

The CSF concentration of LTC4 was significantly higher in healthy control dogs compared to those with SCI. This finding is in contrast to data from a guinea pig spinal cord contusion model, which showed increased parenchymal LTC4 10 min after SCI that persisted 60 min post-SCI. Data from dogs with experimental spinal cord contusion likewise showed abrupt, early increases in LTC4 concentration, measured within the CSF [23]. Our results may have differed from these previous studies for a variety of reasons. First, the median time between SCI and CSF sampling in our population was 36 h; thus, we may not have captured many dogs with post-injury elevations in LTC4. Secondly, a proportion of the naturally injured dogs studied here received either NSAIDs or glucocorticoids, both of which could reduce LT production. Additionally, post-SCI increases in CSF or parenchymal LTC4 may be species-specific, or model-specific. In a study of cats with experimental compressive SCI, LTC4 concentration was not significantly different between sham and SCI animals [28]. Finally, we believe post-SCI shunting within the LT pathway could be possible and might explain the higher CSF LTC4 concentration in control dogs compared to those with injury in this study. For example, macrophages (the predominant inflammatory cell that releases LTC4) that are exposed to a pro-inflammatory environment in vitro have reduced LTC4 synthase mRNA expression [29].

Cerebrospinal PGE2 concentration was significantly increased in dogs with SCI compared to healthy control dogs. Additionally, CSF PGE2 concentration was significantly and positively associated with CSF protein concentration and RBC in injured dogs; both these CSF analytes are increased as a result of intrathecal bleeding and blood-spinal cord barrier disruption [30]. Finally, CSF PGE2 concentration was significantly and positively associated with more severe injury at the time of sampling and also 42-day post-SCI recovery as measured by an ordinal scoring system. The relationship between CSF PGE2 and 42-day post-SCI recovery was assessed using multivariate logistic regression. The strength of this approach is that it takes account of other contributory factors such as immunomodulatory drug administration, initial injury severity, and timing of injury. When we assessed associations between CSF PGE2 and 42-day outcome only using dogs with SCI that had detectable CSF PGE2, the relationships were non-significant. While examining relationships in this manner does eliminate bias from null values, it substantially reduces statistical power (nine dogs assessed). Biologic facets of SCI and recovery are typically investigated in rodent contusion models under a series of highly controlled conditions. Here, we assessed AA pathway metabolites in a naturally-occurring, large animal model of SCI that recapitulates many features of human injury. There are limitations inherent to utilizing samples from dogs with naturally occurring SCI, one of which is the administration of immune response-modulating drugs prior to sample collection. Multivariate logistic regression was used to mitigate influence of administration of these drugs when assessing relationships between CSF AA metabolite concentration and 42-day outcome. We did not, however, directly examine the impact of prior NSAID or GC delivery on CSF AA metabolite concentration in injured dogs. This study could not be adequately powered to investigate the influence of these drugs because of the great variety of interactions between time of injury, time of drug administration, and time of sample collection. Certainly, it is possible that interactions between these drugs and targets in the AA pathway impacted data reported here. Additionally, heterogeneity in injury severity, timing of injury, and vertebral level of compression that are inherent to clinical studies can affect the ability to detect significant inter-group differences. Despite these limitations, this study suggests that lipoprotein associated PLA2, LTC4, and PGE2 are all associated with SCI and may provide information relevant to recovery of function. These data, combined with those from other model systems, provide further evidence that AA metabolites are a viable target for pre-clinical SCI trials.

## Conclusion

We assessed three mediators of AA metabolism in the CSF of dogs with SCI and found evidence that PLA2 and PGE2 concentration is altered during injury and PGE2 concentration is associated with SCI severity and recovery. Previous studies in other neurotrauma model systems likewise have suggested that PLA2 and PGE2 exacerbate SCI. Additionally, we found decreased CSF LTC4 concentration in dogs with SCI compared to healthy dogs, which is contrary to what has been described in experimental models. Interspecies differences in inflammatory networks, variability in injury severity and the anatomic level of injury, and assessment of CSF rather than parenchyma may have influenced the results of this study. Future studies using larger populations of injured dogs to confirm findings in this study and more broadly screen AA pathway constituents might provide valuable information, as could quantification of pathway enzyme mRNA.

## Abbreviations

SCI: spinal cord injury
CSF: cerebrospinal fluid
PLA2: lipoprotein-associated phospholipase A2
LTC4: leukotriene C4
PGE2: prostaglandin E2
IVDH: intervertebral disc herniation
MMP-9: matrix metalloproteinase-9
AA: arachidonic acid
LTs: leukotrienes
PGs: prostaglandins
5-LOX: 5-lipoxygenase
COS: cyclooxygenase
ELISA: enzyme-linked immunosorbent assay
TNCC: total nucleated cell count
NSAIDs: non-steroidal anti-inflammatory drugs
GCs: glucocorticoids
MR: magnetic resonance imaging
CT: computed tomography
MFS: modified Frankel score
TSCIS: Texas Spinal Cord Injury Score
ASIA: American Spinal Cord Association Impairment Score
